# Supplementary figures and images for: Evaluation of five regions as DNA barcodes for identification of Lepista species (Tricholomataceae, Basidiomycota) from China
Source: PeerJ. 2019 Jul 15;7:e7307. doi: 10.7717/peerj.7307 (PMC6637932; doi:10.7717/peerj.7307)

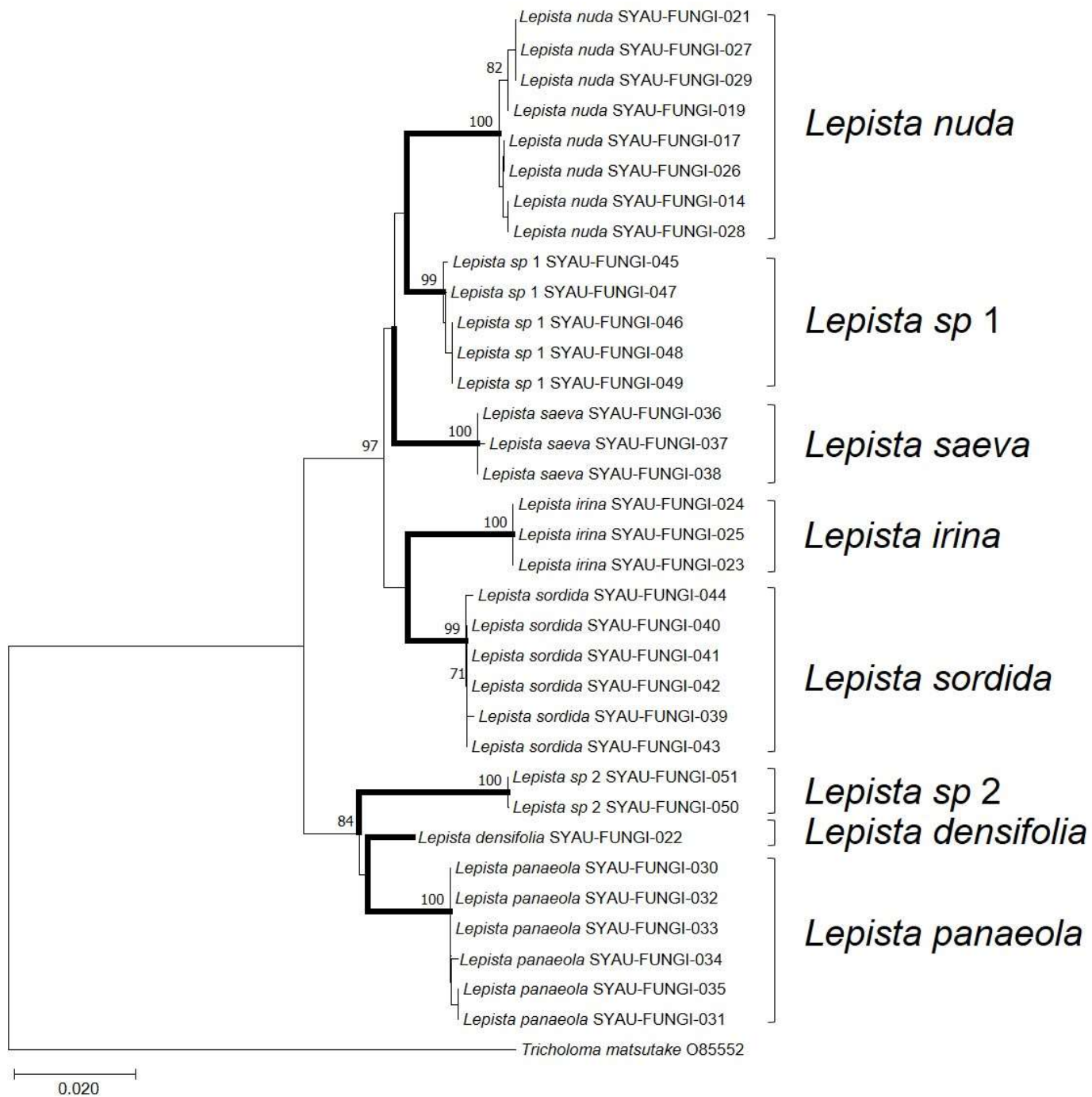

Supplement: Supplemental Information 1 — Figure S1. A neighbor-joining tree generated by analysis of five regions from eight Lepista spp. Bootstrap values ≥70% are shown above the relevant branches. The eight Lepista spp. are highlighted in bold. [file peerj-07-7307-s001.pdf]
